# Editorial: X-raying Zero Hunger (SDG2) targets in Africa and other regions: progress, synergies, opportunities, and challenges, volume II

**DOI:** 10.3389/fpubh.2025.1655507

**Published:** 2025-08-25

**Authors:** Olutosin Ademola Otekunrin, Mojisola Olanike Kehinde, Oluwaseun Ariyo, Barbara Sawicka

**Affiliations:** ^1^Innovation Lab for Policy Leadership in Agriculture and Food Security (PiLAF), University of Ibadan, Ibadan, Nigeria; ^2^Disaster Management Training and Education Centre for Africa, University of the Free State, Bloemfontein, South Africa; ^3^Department of Agricultural Economics, Federal University of Agriculture, Mubi, Nigeria; ^4^Department of Human Nutrition and Dietetics, University of Ibadan, Ibadan, Nigeria; ^5^Department of Plant Production Technology and Commodities Science, University of Life Science in Lublin, Lublin, Poland

**Keywords:** SDG 2, food security, undernourishment, food access, violent conflict, Africa

## 1 Introduction

This editorial introduces Volume II of a Research Topic, “*X-raying Zero Hunger (SDG2) Target in Africa and Other Regions: Progress, Synergies, Opportunities, and Challenges*” following the initial 2024 publication. Volume I, comprised of eight articles and forty-four contributors from fifteen countries, provided a comprehensive assessment of the world's progress toward Sustainable Development Goal 2 (Zero Hunger). The original research explored the advancements, interdependencies between SDG2 and other SDGs, and the specific opportunities and challenges faced by Africa and other regions in attaining Zero Hunger by 2030. Volume II seeks to build upon this foundation, continuing the vital conversations initiated in the first volume to further investigate and address the complexities surrounding global food security.

Hunger is not simply a fleeting crisis confined to specific regions or moments in time; it is rapidly becoming a permanent and a deeply entrenched problem on the lives of millions globally, as presented by António Guterres, the United Nations Secretary General in the 2025 Global Report on Food Crises (1). Driven by the converging forces of conflict, escalating geopolitical tensions and climate change impacts, underlying environmental vulnerabilities, and widespread economic instability, food and nutrition crises are no longer temporary setbacks. Instead, they are defining the life experiences of countless individuals, not just for weeks or months, but for years, and in some cases, for entire lifetimes, perpetuating cycles of deprivation and suffering (1).

The report paints a concerning picture of escalating global hunger, noting that over 295 million individuals experienced acute food insecurity in the past year—a distressing trend that has continued for six consecutive years. Driven by the devastating combination of conflict, socioeconomic shocks, and environmental crises, catastrophic hunger is reaching unprecedented levels in regions spanning from Gaza and Sudan to Yemen and Mali, pushing vulnerable households to the very edge of survival. Displacement, triggered by violence and climate-related disasters, is further exacerbating the crisis, as families are uprooted from their homes and exposed to heightened risks of malnutrition and mortality. Compounding these challenges, increasingly severe climate extremes are wreaking havoc on global food systems, devastating crop yields, and disrupting critical supply chains, further jeopardizing food security for millions worldwide (1–3).

Sustainable Development Goal 2 (SDG 2), also known as Zero Hunger, aims to achieve a world free from hunger by 2030. As one of the 17 SDGs, SDG 2 seeks to motivate member countries to eliminate hunger, ensure food security, improve nutrition, and promote sustainable agriculture (4–7).

According to the 2022 Global Food Security Index (GFSI), seven of the top ten countries with the highest food security scores are in Europe, two are in North America (Canada and the United States), and one (Japan) is in the Asia-Pacific region. In contrast, sub-Saharan Africa faces some of the highest rates of food insecurity globally, with a significant proportion of the population unable to access nutritious diets. The percentage of people experiencing severe food insecurity in Africa increased from 17.2% in 2015 to 24.0% in 2022, exceeding the global rate of 11.8% and surpassing that of any other region (8, 9).

**Figure 1:**
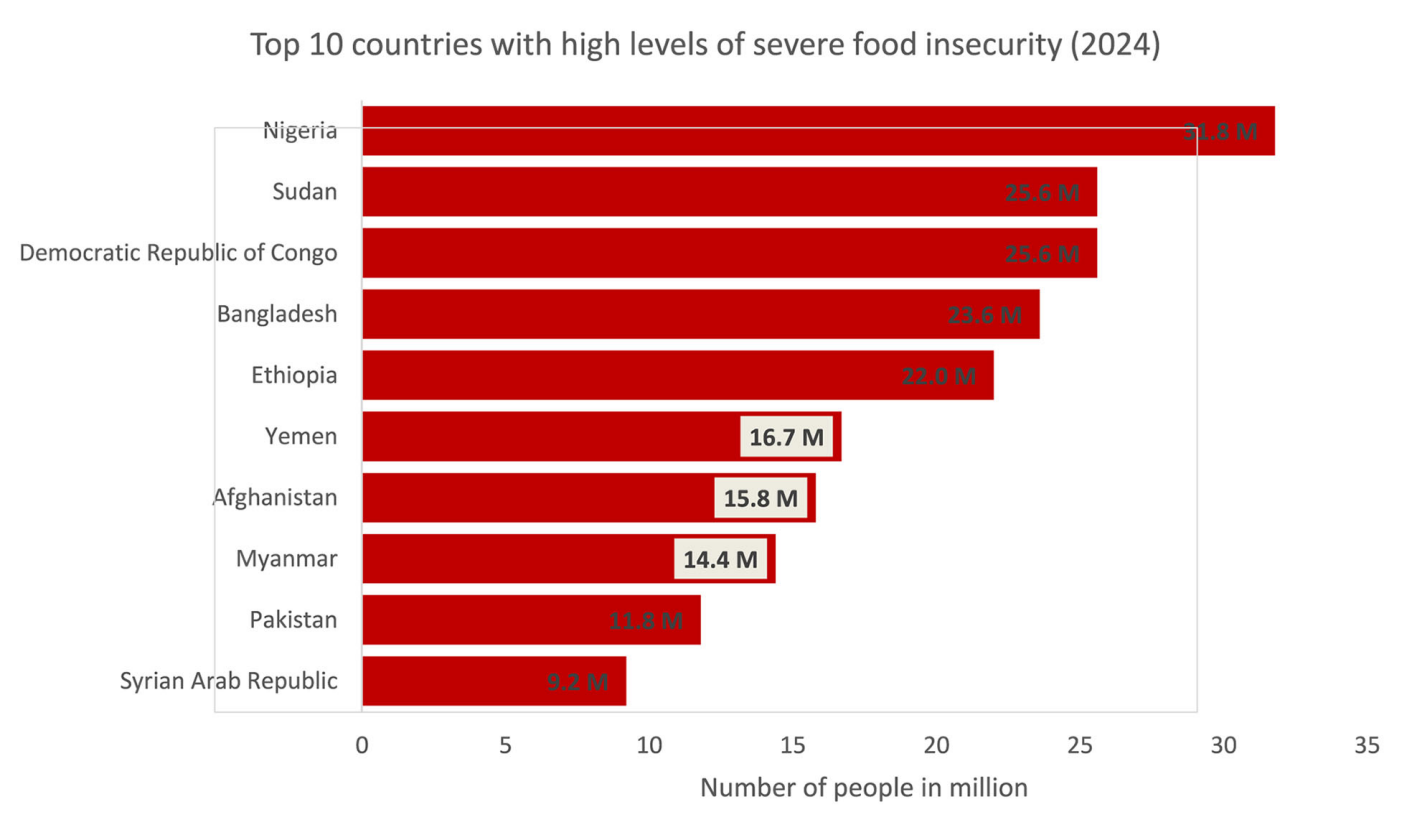
Top 10 countries with the highest number of population facing severe food insecurity in 2024. Source: Authors' compilation using 2025 GRFC (1).

Furthermore, data from the 2025 GRFC revealed that Nigeria (31.8 million), Sudan (25.6 million), and the Democratic Republic of the Congo (also 25.6 million) rank among the top three countries with the highest populations facing severe food insecurity in 2024 (see Figure 1). These three countries collectively account for 42% of the total population in the top ten most affected nations, highlighting that Africa bears the highest burden of global hunger and food insecurity (1).

## 2 Articles in the Research Topic volume II

This Research Topic volume II presents nine articles that have successfully undergone rigorous peer review, meeting the high standards of Frontiers in Public Health. While these articles explore diverse topics, theoretical perspectives, and methodologies, they are all connected to the overarching themes of this RT volume II.

Gujo and Modiba investigated food insecurity in the South Omo Zone of Southern Ethiopia, focusing on pastoralist and agrarian communities. Despite existing interventions, food insecurity remained a significant issue with limited data specific to the region. The study aimed to determine the prevalence of food insecurity and identified contributing factors. A cross-sectional study was conducted with 597 participants from randomly selected households. The Household Food Insecurity Access Scale (HFIAS) was used for measurement, and data were analyzed using SPSS V25. Binary logistic regression identified factors linked to food insecurity (*p* < 0.05).

The study found that 42.2% of households experienced food insecurity, with varying severity levels. Key factors impacting food security included female-headed households, high dependency ratios, lack of maternal education, absence of participation in safety net programs, and lack of land ownership. These findings highlight the need for targeted interventions in the South Omo Zone. The study recommends improving female education, advancing agricultural techniques, promoting family planning, and expanding safety net programs to enhance food security and community wellbeing.

Tamir et al.'s study, titled “*Spatial heterogeneity and predictors of stunting among under five children in Mozambique: a geographically weighted regression,”* examines the spatial variations and predictors of stunting in Mozambican children under five, using Geographically Weighted Regression (GWR) to account for localized relationships. The study included a sample of 3,910 under-five children. Data from the 2011 Mozambique Demographic and Health Survey (DHS) were analyzed, incorporating variables like household wealth, maternal education, sanitation access, and healthcare access. GWR's performance was compared to Ordinary Least Squares (OLS) regression, highlighting the benefits of considering spatial heterogeneity. The analysis pinpointed areas where specific predictors, like maternal education and sanitation, had a stronger influence on stunting.

The findings revealed significant spatial variations in stunting predictors across Mozambique. The impact of maternal education and sanitation access varied geographically, emphasizing the importance of local context in intervention design. The study concludes that geographically targeted interventions, informed by GWR-identified spatial patterns, are more likely to effectively reduce stunting among under-five children in Mozambique.

Adugna et al.'s explores the nutritional wellbeing of 309 school children (6–14 years old) in Addis Ababa, Ethiopia, considering socioeconomic, environmental, and behavioral influences. Acknowledging that poor nutrition impacts development and learning, the research sought to pinpoint key factors to guide effective interventions. The study offers valuable insights into the multifaceted influences on child nutrition within an urban environment. To achieve this, data was gathered on height and weight, household socioeconomic status, environmental conditions like sanitation and water access, and behaviors including diet and physical activity. Statistical analyses determined the prevalence of undernutrition (stunting, underweight, thinness) and identified significant predictive factors.

The results indicated a notable prevalence of suboptimal nutrition among Addis Ababa's school children. Key predictors of undernutrition included low socioeconomic status, inadequate sanitation and clean water, poor dietary diversity, and limited physical activity. The study emphasizes the necessity of comprehensive, multi-sectoral interventions addressing socioeconomic and environmental factors, alongside promoting healthy eating and active lifestyles.

Beni et al. explores quantile regression to pinpoint the key factors driving malnutrition in Gabon, Gambia, Liberia, Mauritania, and Nigeria. Recognizing that these factors may impact children differently depending on their nutritional status, the researchers analyzed influences across the spectrum of malnutrition, rather than just looking at average effects. This provides a more detailed understanding of the complex causes of malnutrition in the region. Data from Demographic and Health Surveys (DHS) in these five countries were analyzed, focusing on under-five children. The study examined socioeconomic, demographic, and health variables as potential drivers of malnutrition, measured by stunting, wasting, and underweight. Quantile regression models estimated the impact of these variables at different points (quantiles) of the malnutrition indicators.

The results showed that the drivers of malnutrition varied significantly across different nutritional status levels. For instance, maternal education and household wealth had a greater impact on improving nutrition for children with the poorest nutritional status compared to those already better nourished. The study emphasizes the need for customized interventions that address the specific needs of children at different levels of malnutrition and concludes that quantile regression is a useful tool for identifying effective, targeted strategies to fight malnutrition in West Africa.

Endawkie et al.'s study investigates disparities in inadequate minimum dietary diversity (MDD) between poor and non-poor children aged 6–23 months in Sub-Saharan Africa. Recognizing that adequate dietary diversity is essential for proper growth and development, the researchers aimed to identify factors contributing to the MDD gap between these groups. They employed multivariate decomposition analysis to assess the influence of various socioeconomic and demographic factors on this disparity. Data from DHS conducted across multiple Sub-Saharan African countries were analyzed, focusing on children aged 6–23 months. Key variables included household wealth, maternal education, access to healthcare, and geographic location. This decomposition method quantified how much these factors explain the observed differences in inadequate MDD between poor and non-poor children.

The findings revealed a significant disparity in inadequate MDD, with poor children disproportionately affected. The analysis identified household wealth, maternal education, and healthcare access as major contributors to this gap. The study underscores the need for targeted interventions addressing these socioeconomic inequalities to improve dietary diversity and nutritional outcomes for all children, especially those from disadvantaged backgrounds.

Gashe et al.'s study titled “*Investigating a severe acute malnutrition outbreak in Dubti District, Awsiresu Zone, Afar Region, Northeast Ethiopia (2022)”* examines the causes and factors contributing to a severe acute malnutrition (SAM) outbreak in the Dubti District of Northeast Ethiopia. The study investigates the epidemiology of the outbreak, identifies key risk factors, and assesses the affected population's characteristics. By analyzing data collected during the 2022 outbreak, the researchers aimed to understand the underlying drivers such as food insecurity, inadequate healthcare access, poor sanitation, and socio-economic conditions. The findings highlight critical gaps in nutrition and public health interventions in the region, emphasizing the need for targeted strategies to prevent future outbreaks. The study calls for strengthened community-based nutrition programmes, improved healthcare delivery, and multisectoral collaboration to address the root causes of malnutrition in this vulnerable population.

Tebeje et al.'s study, titled “*Minimum meal frequency and associated factors among children aged 6–23 months in Sub-Saharan Africa: a multilevel analysis of the demographic and health survey data,”* examines the prevalence of minimum meal frequency (MMF) among young children in Sub-Saharan Africa and the factors that influence it. Using data from DHS across multiple countries, the study employs multilevel analysis to explore individual, household, and community-level determinants affecting whether children aged 6–23 months receive the recommended minimum number of meals per day. The findings reveal significant regional variations in MMF and identify key predictors such as maternal education, household wealth, access to healthcare, and rural vs. urban residence. The study emphasizes the need for targeted nutrition interventions and policies addressing these factors to improve feeding practices and ultimately enhance child health and development across Sub-Saharan Africa.

Tareke et al.'s article titled “*Identifying high-risk population segments for underweight, overweight, and obesity among reproductive-age women in sub-Saharan Africa”* explores the dual burden of malnutrition among 247,911 reproductive-age women aged 15–49 in 33 sub-Saharan African countries. Using DHS data, the study reports that 11% of women are underweight, 18% overweight, and 10% obese. These findings highlight the complex and shifting nature of nutritional challenges in the region. The study identifies underweight as more common among younger, poorer, rural women with limited education or media exposure. Conversely, overweight and obesity are linked to older age, urban residence, and higher socioeconomic status, use of modern contraceptives, higher parity, and lack of breastfeeding. Employment appears to offer protection across all malnutrition categories, suggesting economic empowerment may enhance health outcomes.

The authors recommend targeted public health strategies: addressing undernutrition among younger, rural, and low-income women, and focusing obesity and overweight interventions on older, wealthier, urban populations—particularly in southern and central Africa. The study emphasizes the need for region-specific, socially informed nutrition policies to combat malnutrition's contrasting trends.

The work of Eyasu et al. titled “*Impact of crop commercialization on multidimensional poverty in rural Ethiopia: propensity score approach”* examines how market-oriented crop production influences rural household wellbeing. Using data from the 2018/19 Ethiopian Socioeconomic Survey, the study analyzed 2,714 rural households across 59 administrative zones. The researchers constructed a Rural Multidimensional Poverty Index (R-MPI) based on five key dimensions: nutrition and health, education, living standards, rural livelihoods, and risk exposure. They employed a generalized linear mixed-effects model and propensity score methods to assess the causal relationship between crop commercialization and multidimensional poverty. Findings reveal that 47.8% of rural households were multidimensionally poor, with the highest deprivation occurring in living standards and nutrition. Spatial disparities were evident, with poverty concentrated in zones like Shebelle, Konso, and regions such as Afar and Somali. Importantly, households engaged in crop commercialization were significantly less likely to experience multidimensional poverty. The odds of being multidimensionally poor were reduced by ~21% among commercializing households compared to their subsistence counterparts.

The study concludes that crop commercialization is a vital pathway to reducing multidimensional poverty in rural Ethiopia. To maximize its benefits, the authors recommend targeted policies that support smallholder market access, infrastructure development, and region-specific interventions. Tackling spatial poverty hotspots is essential for inclusive and sustainable rural development.

## 3 Conclusion

This Research Topic brings together nine scholarly articles that collectively address a vital and underexplored area in the existing literature: the global fight against hunger, with a dedicated focus on the African continent. These articles provide an in-depth examination of the progress made so far, critically assessing where efforts have succeeded and where significant gaps remain. A central theme running through the Research Topic is the interplay between Sustainable Development Goal 2 (Zero Hunger) and other interconnected SDGs—such as poverty reduction, health, climate action, and sustainable agriculture—underscoring the need for integrated, cross-sectoral strategies. The research further unpacks the specific challenges faced by African nations, including food system vulnerabilities, policy implementation barriers, limited financing, and institutional weaknesses. At the same time, it explores the opportunities presented by innovation, regional cooperation, and targeted social protection programmes. By analyzing the design, execution, and effectiveness of relevant plans and interventions, the articles offer valuable insights into both systemic issues and localized solutions.

Overall, this body of work contributes significantly to understanding the complexity of achieving Zero Hunger by 2030, offering evidence-based recommendations and fresh perspectives that can inform policy, practice, and future research aimed at creating a hunger-free world.
